# Editorial: Emerging technologies for assistive robotics: current challenges and perspectives

**DOI:** 10.3389/frobt.2023.1288360

**Published:** 2023-10-10

**Authors:** Lyuba Alboul, Maya Dimitrova, Anna Lekova, Vassilis George Kaburlasos, Peter Mitrouchev

**Affiliations:** ^1^ Industry and Innovation Research Institute, Sheffield Hallam University, Sheffield, United Kingdom; ^2^ Institute of Robotics, Bulgarian Academy of Sciences (BAS), Sofia, Bulgaria; ^3^ HUMAIN-Lab, Department of Computer Science, International Hellenic University (IHU), Kavala, Greece; ^4^ Université Grenoble Alpes, Saint Martin d’Hères, France

**Keywords:** assistive robotics, healthcare, social robots, frailty, humanoid robots, cyber-physical systems, human-robot interactions

## 1 Introduction

Assistive robotics is an emerging research and practical field, mostly oriented toward healthcare and support for people with special needs. In a broader sense, however, it can be defined as robotics tools and devices to provide users of all abilities and needs with physical, cognitive, and social assistance. In principle, robots can be introduced successfully at work and other places such as hospitals, care homes, and people’s homes, and are able to interact directly with humans—a Research Topic of intensive research in various recent research projects, funded nationally or internationally^1^ ^2^ ^3^ ^4^. The role of assistive robotics has also increased due to the COVID-19 pandemic (Peter et al., 2022). Our understanding is that robots are best employed in supporting the professional role of the human, especially in healthcare.

The Research Topic of the current is to address the new trends and emerging technologies in assistive robotics. One of the focus of the published articles is on the design of robots capable of assisting humans on various levels of social, medical, and biomedical analysis. A special emphasis is on the notion of “emergence”^5^ since technologies develop constantly and change their place in the utility-scale of the individual (Pessa, 2002, p.379). For example, a telephone today is of different priority and an accessory for support of daily living than it used to be; it can monitor the indicators of a person’s life, remind them of the scheduled tasks or pills to take, and *finally*, help them get in contact with their relatives.

## 2 Overview of the articles

When robotics is discussed for individual care, it is important to investigate what currently is being understood by “assistance”. The paper by Saille et al. systematically addresses the Research Topic from an “emergence” perspective. The authors developed a comparative set of focus group workshops using Community Philosophy, LEGO^®^ Serious Play^®^, and Design Thinking to explore how people with a range of different physical impairments used these techniques to envision a “useful robot.” The outputs were then analysed by a group of roboticists and designers to explore how they interacted with the thematic map produced by the users. The three user workshops elicited a synthesised view of the lived experience of disability, experiences of care, expected characteristics of a desirable care robot, and tensions in each of these categories of consideration. One of the outcomes of the study was a list of 31 relevant themes identified by the workshops, addressing the roles of the robot in the life of people with disabilities. It is interesting to see which of these received the highest scores since after some time the priorities may change and a novel theme hierarchy may emerge.

Users with special needs imagine that the useful robot helps with physical tasks (16)^6^, monitors “me” (7), helps overcome sensor difficulties (i.e., physical cognitive impacts) (7), is activated by voice (7), alleviates the burden of management (6), and is user co-created (5).

Therefore, the term “assistive robotics” is defined comprehensively as robots that coordinate their actions to provide their users with physical, cognitive, and social assistance. Our aim is to consider a wide community of users, including users of all abilities and needs.

In terms of physical assistance, an important aspect is the design of novel orthotic devices to assist post-stroke patients in performing everyday tasks to support their independent living. The paper of Salvietti et al. presents a novel control system for compensation of grasping, which was tested by three patients. Although based on a limited sample, extensive analysis is provided on the potential of the device to support post-stroke rehabilitation.

To compensate for the lack of a functional arm and hand, a wearable system that combines different assistive technologies including sensing, haptics, orthotics, and robotics is designed. The result is a device that helps lift the forearm by means of a passive exoskeleton and improves the grasping ability of the impaired hand by employing a wearable robotic supernumerary finger. The pilot study was conducted to test the capability of the device to assist in performing daily living activities, confirm its usefulness, and serve as a first step in the investigation of novel paradigms for robotic assistance.

Concerning the “useful robot,” the second place of the themes’ hierarchy is the ability of the robot to *monitor* the user, which may be perceived as unexpected because of the present-day concerns about privacy when interacting with intelligent technology. Yet, one specific approach towards deployment of the ability to monitor, diagnose the mental state, and make decisions about a treatment strategy in healthcare is described in the paper of Salhi et al. “*Towards Robot-Assisted Therapy for Children with Disabilities -The Ontological Knowledge Models and Reinforcement Learning-based Algorithms*.” The automatic process of diagnosing autism at the present moment may still seem unrealistic, yet the proposed modelling and simulation approach comes to fulfil the finding in the study of Salhi et al. which focuses on the need for cognitive support in perceiving and understanding the surrounding reality as well as for acting in the world by the users.

The research focused on robot-assisted therapy and introduced a humanoid social robot in a paediatric hospital care unit. By analysing many aspects of the child’s behaviour, such as verbal interactions, gestures, and facial expressions, the robot was able to diagnose the child’s condition. The robot could reproduce consistent experiences and actions for children with limitations of their communication capacity, such as in cases of autism. A novel approach based on deep learning and reinforcement learning algorithms was proposed, which was supported by an ontological knowledge base that contained relevant information and knowledge about patients, screening tests, and therapies. A humanoid robot NAO was used to assist the therapist by equipping the robot with the ability: 1) to detect whether a child has autism or not, using a convolutional neural network, 2) to recommend a set of therapies based on a selection algorithm using a correspondence matrix between screening test and therapies, and 3) to assist and monitor children with autism by executing tasks that are required by those therapies.

In the paper of Dimitrova et al. “*A Review of Possible EEG Markers of Abstraction, Attentiveness, and Memorisation in Cyber-Physical Systems for Special Education*,” the “useful robot” plays the role of a co-teacher by mimicking the human way of being a skilful teacher—socially and pedagogically. The paper explores the ‘emergence’ of novel concepts, memories, and ideas during the lesson by focusing on the underlying brain mechanisms of learning in inclusive classes. It focuses on the available knowledge of possible EEG markers of abstraction, attentiveness, and memorisation related to predicting effective mental and brain processing during the lesson. The role of processing *abstraction* is emphasised as the learning mechanism, which is given priority over the other mechanisms by the cognitive system. The description of the EEG effects is accompanied by the analysis of some implications for the design of novel educational scenarios in inclusive classes being valid for life-long learning as well.

Overall, this Research Topic provides some novel insights into the topics of human wellbeing, confidence, and education via technologies with emergent properties. It is in line with the present-day understanding of disability as a discrepancy between the goal of the person and the available means (technological, social, or motivational) for gaining it, i.e., “Disability in an AfA^7^ context is not a personal trait but a consequence of the relationship between the user and their resource system” (IMS AccessForAll, 2022 ^8^). The current Research Topic provides an original view of the technological, cognitive, and neurocognitive interactions with an assistive robot for better achievement of one’s personal goals.

## Footnotes

1
https://www.emergencerobotics.net

2
https://robomed.bg/

3
https://cordis.europa.eu/project/id/777720

4
https://www.nrc.go.kr/eng/main.do

5 “the emergence can be defined only *relatively to a given observer”*.

6 The numbers in brackets are the instances of the theme being formulated by the users.

7 Access for all.

8
https://www.imsglobal.org/accessibility/accdrdv2p0/html/ISO_ACCDRDv2p0_InfoModelv1.html.
